# Warming Up Body and Mind: Combined Cognitive and Exercise Priming Improves 1‐Mile Time Trial Performance in Recreational Runners

**DOI:** 10.1002/ejsc.70163

**Published:** 2026-04-15

**Authors:** Hannah Mortimer, Neil Dallaway, Jesús Díaz‐García, Christopher Ring

**Affiliations:** ^1^ School of Sport, Exercise & Rehabilitation Sciences University of Birmingham Birmingham UK; ^2^ Faculty of Sport Sciences University of Extremadura Badajoz Spain

## Abstract

Combined cognitive and physical warmups can improve sport and exercise performance more than standard physical only warmups. The effects of combined warmups on running have yet to be determined. Accordingly, the aim was to compare the effects of combined and standard warmups on 1‐mile time trial performance. Experienced recreational runners (11 males, 14 females) completed three counter‐balanced sessions. In each session, they completed one of three warmups (physical only, physical plus low cognitive load, physical plus high cognitive load) immediately followed by four laps of a 400 m outdoor track. The physical warmup comprised three activities: 1200 m easy jog, 800 m alternating jogs and strides and 3‐min active stretching drills. The combined warmups required completion of four easy/hard 3‐min cognitive tasks before and after the physical activities. We measured readiness (after warming up), perceived exertion, heart rate and running kinematics (while running) and fatigue (after running). ANOVAs showed that compared to the standard physical warmup both combined warmups improved running times by 8–11 s (2%–3%), increased readiness to perform, reduced perceived exertion, lowered heart rate and reduced perceived mental fatigue. Combined warmups with interleaved short cognitive and exercise tasks improved 1‐mile time trial performance. This study provides evidence that combined warmups prime athletes to run faster by increasing readiness to perform and reducing perceived and actual effort.

## Highlights

- Intermixing brief executive‐function tasks within a standard physical warm‐up improved 1‐mile time trial performance by 2%–3% in recreational runners.
- Combined cognitive–physical warm‐ups increased readiness to perform ratings while reducing perceived exertion and average heart rate during running compared to a standard physical warm‐up.
- Both low and high cognitive load conditions enhanced performance, indicating short cognitive priming may optimise endurance preparation. Future research should continue to explore this.

## 1 Introduction

Athletes warmup to increase body temperature, cause neuromuscular activation, reduce injury and improve readiness for subsequent training or competition (Afonso et al. 2024; Pereira et al. 2025). Warmup protocols initially relied on trial‐and error based on athlete and coach experiences, however, more recently empirical research has explored the physiological mechanisms that underpin performance gain following warmups (McGowan et al. 2015). Completing a warmup can improve performance in sport (e.g., Fradkin et al. 2010), including running (e.g., Paris et al. 2021; Alves et al. 2023). For instance, moderate to high intensity warmups improved 800 m running performance by 2%–4% in student athletes compared to low intensity warmup (Keesling et al. 2022) and high intensity warmup enhanced 5000 m running performance more than low intensity warmup (Alves et al. 2023).

Warmup guidelines frequently mention the enhancement of the athlete's cognitive state, including preparation for action and neuromuscular activation (Afonso et al. 2024). However, there is limited evidence to support these claims. Short, structured warmups have been shown to improve readiness to performance and lower rating of perceived exertion (RPE) in maximal sprint performance (Van den Tillaar et al. 2025) and increase preparatory arousal prior to strength performance (Perkins et al. 2001). Anecdotal evidence reveals that athletes complete cognitive activities prior to competition (e.g., incorporating reaction time and decision‐making tasks) designed to improve information processing and activate the central nervous system (Sequeira and Bhataiya 2021; Ezhov and Zakharova 2024). Due to the high levels of cognitive processing to maintain effort, pacing and tactical decision making, athletes often use imagery as a cognitive component of the warmup for arousal regulation and cognitive modification (Jones and Stuth 1997) and therefore it has been suggested that short cognitive tasks could result in compensatory cerebral blood flow which could improve performance (N. P. Dallaway 2019).

Athletes and coaches seek ways to improve readiness to perform in training and competition, including activities that enhance mental preparedness and subsequent performance (Valcarce‐Merayo and Latella 2023). Nonetheless, research on the effects of cognitive warmups on sport and exercise performance remains limited, particularly regarding the potential role of priming cognitive operations.

Cognitive executive functions are key processes underpinning behaviour that support attentional control, goal‐directed actions, self‐regulation and cognitive flexibility (Diamond 2013). The neuropsychological literature has established that certain cognitive tasks target executive functions, shown by activation of the dorsal anterior cingulate cortex, which plays a role in cognitive processing and priming (Bush and Shin 2003). Athletes typically display superior cognitive performance (including executive function) compared to non‐athletes, with the performance advantage increasing as a function of sporting experience (Ren et al. 2025). This suggests the possibility that activating cognitive processes using classic cognitive tasks during warmup may cognitively prepare athletes and thereby enhance subsequent performance. However, the current evidence gap requires research to better understand this phenomenon.

Research has shown that cognitive tasks, designed to prime the performer, can subsequently improve performance of videogames (Oei and Patterson 2014) and cognitive tasks (Lemaire and Brun 2018; Wexler et al. 2016). Recent studies show that combining cognitive and physical warmup activities can improve subsequent sport performance. A study found that basketball players' dribbling performance was improved following completion of a 10‐min cognitive task and a standard physical warmup (González‐Fernández et al. 2024). Similarly, studies found that adding short cognitive tasks to standard physical warmup protocols further improved sport and exercise performance (Díaz‐García et al. 2025). Specifically, one study found that combined warmups improved padel shot accuracy more than standard physical warmup in young adult padel players. The second study found that combined warmup improved aerobic (walking time) and muscular (bicep curls and squats) endurance relative to no warmup and physical only warm‐up in sedentary older adults. In both studies, short‐to‐medium duration cognitive tasks were intermixed with physical warmup drills. Interestingly, an optimal (or ‘just right’) cognitive task duration was found, with short‐to‐medium cognitive tasks improving but long cognitive tasks impairing sport and exercise performance. Endurance running performance is influenced not only by physiological capacity but also by cognitive and perceptual processes such as attentional focus, pacing regulation and the tolerance of effort‐related sensations (Marcora et al. 2009; Pageaux 2014). While there is not any direct evidence on cognitive warm ups and endurance exercise at present, research on mental fatigue and Brain Endurance Training has shown that prior cognitive activity can influence perceived exertion and endurance performance across exercise modalities, including running (Van Cutsem et al. 2017). Continuous middle‐distance running requires sustained cognitive engagement to regulate pace under increasing fatigue, suggesting that cognitive priming during warmup may enhance readiness to perform and reduce perceived effort during a hard, continuous time trial. This highlights a dose‐response relationship and justifies exploring both the intensity and duration of the cognitive warmup tasks in running performance.

Completing brief cognitive tasks between bouts of exercise has been suggested to induce a flow state (Rautu et al. 2025). The experience of flow is associated with improved physical performance (Csikszentmihalyi 2000), including sport (Jackson et al. 2001). Flow during cognitive tasks has been associated with increased neural activity in the left anterior inferior frontal gyrus and left putamen (Ulrich et al. 2014) and increased frontal theta activity, with right central alpha activity increasing in line with task difficulty (Katahira et al. 2018). In sum, cognitive tasks may benefit subsequent athletic performance if they are able to engage relevant brain areas and pathways alongside physical exercise tasks. Accordingly, a combination of cognitive and physical activities during a warmup may improve subsequent physical performance by mentally and physically preparing (i.e., priming) the athlete for the upcoming psychological and physiological demands of sport and exercise tasks.

In sum, preparation strategies are critical for optimal athletic performance, however, the key physical and cognitive components of warmups and their effects on subsequent performance are not yet fully understood. Combined warmups may offer performance benefits and have valuable practical applications to the field by enhancing readiness to perform and physical performance in both training and competition. The current study therefore aimed to explore this priming effect in running time trial performance. The first study purpose was to compare the effects of combined warmups versus physical only warmup on running performance. Specifically, it examined the effect of cognitive task intensity on performance by having runners complete easy or hard cognitive tasks before and after standard physical warmup activities. It was hypothesised that running times would be shorter with combined warmups than physical warmup and that running times would be shortest with hard cognitive tasks compared to easy cognitive tasks. The second study purpose was to evaluate the effects of warmups on psychobiological processes. It was hypothesised that combined warmups would mentally prime runners and improve their running efficiency.

## 2 Method

### 2.1 Participants

Twenty‐five (11 males, 14 females) recreational runners, aged 19–37 (*M* = 28.36, SD = 4.13) years, were recruited from the London running community club network and provided informed consent. Participants had 4.04 (SD = 2.83) years running experience, had an average mile personal best time of 6 min 47 s (*M* = 407, SD = 98 s) and 5 km personal best of 23 min 31 s (*M* = 1411, SD = 167 s). In the 3 months prior to testing, participants ran 30.28 (SD = 15.89) km per week. Inclusion criteria required participants to be injury free and have access to an iPhone and a Garmin running watch. The protocol was approved by the Ethics Committee at the University in accordance with the Declaration of Helsinki. Participants were naïve to the study purposes but were informed they would complete a series of physical and cognitive tasks. Power calculations indicated that with a sample size of 25, the study was powered at 80% to detect significant (*p* < 0.05) within‐participant effects corresponding to a small‐medium effect size (*n*_*p*_^2^ = 0.06, *f* = 0.26) using contrast analyses.

### 2.2 Study Design and Procedure

The study employed a within‐participant experimental design to assess the effects of priming on performance and process measures. Participants completed three warmup protocol sessions with the lead researcher: physical only priming, low cognitive load priming plus physical priming and high cognitive load priming plus physical priming. Each warmup was immediately followed by a 1‐mile time trial on a track (four 400 m laps). Testing order was counterbalanced across participants.

**FIGURE 1:**
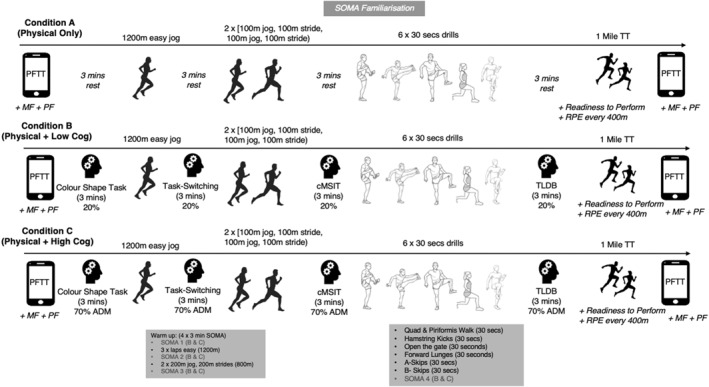
Study protocol for each warmup condition.

Prior to each testing session, participants were instructed to aim for a good night's sleep (7–9 h), avoid alcohol for 48 h, avoid non‐habitual vigorous exercise for 24 h and avoid intense running or heavy resistance lower body exercise for 48 h. They were instructed to avoid caffeine for 3 h and refrain from eating for 1 h before the start of each session. To ensure consistency across sessions, participants were asked to fuel sessions in the same way (e.g., same mealtimes and foods), to hydrate in the same manner and to wear the same running trainers (non‐carbon or nylon plated) and training clothing. Testing was conducted at the same time of day and at least a 48‐h recovery period separated sessions. Participants completed three sessions within a 7–14 day period to limit any potential adaptations from their own training that could impact performance. The three testing sessions followed the format outlined in Figure 1 where 3‐min rest periods or cognitive tasks separated the physical warmup activities. The measures and timepoints are detailed below.

### 2.3 Measures and Tasks

#### 2.3.1 Ratings

##### 2.3.1.1 Mental Fatigue

Mental fatigue was assessed subjectively and objectively at the start (baseline) and end of each session (post time trial). Subjective mental fatigue was assessed by rating perceived mental fatigue on a 10‐point Likert scale ranging from 1 (*no mental fatigue*) to 10 (*maximal mental fatigue*) (Mortimer et al. 2024). Objective mental fatigue was assessed behaviourally using a 90 s Psychomotor Fatigue Threshold Test (PFTT) (Rautu et al. 2025) which was administered via the SOMA‐NPT application (SOMA Technologies n.d.) running on an iPhone. Participants were presented with a series of circles (10 green, 10 red and 5 yellow) on the screen. They responded as quickly and accurately as possible by pressing the left or right button on the screen when they saw a green or red circle, respectively. They did not respond when they saw a yellow circle. Mean reaction time was computed as an objective index of mental fatigue.

##### 2.3.1.2 Physical Fatigue

Physical fatigue was assessed using a 10‐point Likert scale ranging from 1 (*no physical fatigue*) to 10 (*maximal physical fatigue*) at start (baseline) and end of each session (post time trial).

##### 2.3.1.3 Readiness to Perform

Participants reported their perceived readiness to perform on a 10‐point scale ranging from 1 (*not ready to perform at all*) to 10 (*extremely ready to perform well*) immediately before beginning the time trial. Similar self‐assessment approaches have previously been used to track athletes’ psychological and physical preparedness and can offer insight into performance states (e.g., Saw et al. 2015).

#### 2.3.2 Cognitive Priming

Prior to testing, participants completed four 1‐min familiarisation tasks on the SOMA‐NPT app (SOMA Technologies, Switzerland) on a smartphone. Four different cognitive tasks were used in the combined warmup protocols to engage higher order executive functions (see Table S1: Table 1). The colour‐shape task (*task switching*), Time‐Load Dual Back (TLDB; *memory updating, task switching, response inhibition*), Colour Multi‐Source Interference Task (CMSIT; *response inhibition*, *cognitive interference and decision making*) and Task Switching (*task switching*) were used. The 3‐min tasks were intermixed with physical tasks and drills in the combined warmup protocols. In the low combined condition, tasks were set at a starting intensity of 20% throughout the 3 min. In the high combined condition, tasks were set at a starting intensity of 70% and programmed in adaptive mode, whereby task difficulty varied with performance to maintain cognitive load (O’Keeffe et al. 2019). Specifically, the inter‐stimulus interval for the next block of 10 trials was shortened if performance accuracy of the current block of 10 trials was equal to or greater than 90% correct; and was lengthened if performance accuracy of the current block of 10 trials was equal to or less than 80% correct. This has been shown to maintain intensity and cognitive engagement (Mortimer et al. 2024).

#### 2.3.3 Physical Priming

The sessions took place on a 400 m polymeric track. Participants used the inside lane of the track. The physical components of the warmup were the same for all three priming conditions. Participants completed three laps (1200 m) of easy jogging, two laps (800 m) of 100 m jog followed by 100 m of strides and a series of six 30 s drills designed to engage and warmup running‐specific muscles (see Figure 1).

#### 2.3.4 Time Trial

Running performance was measured using a 1‐mile time trial (i.e., four continuous laps of the track). Participants were instructed to run in the best time they could and informed that it should be very physically demanding, allowing participants to decide how best to run the distance. To eliminate real‐time performance feedback, the face of the Garmin smartwatch was covered throughout the mile, allowing runners to rely solely on perceived exertion and ‘feel.’ Feedback has been found to mitigate the effects of cognitive tasks on endurance performance (N. Dallaway et al. 2022). Mile time was recorded in seconds.

#### 2.3.5 RPE

After each lap (400 m), participants reported their Rating of Perceived Exertion (RPE; Borg 1998) on a scale ranging from 0 (*minimal exertion*) to 10 (*maximal exertion*). For analysis, mean RPE across the four laps was calculated for each participant and condition.

#### 2.3.6 Sleep and Menstrual Cycle Tracking

The sleep score from the participant's Garmin watch, for the previous night, was taken each session as well as menstrual day and cycle phase, where applicable, to control for these variables and ensure consistency where possible.

#### 2.3.7 Running Data

A wearable Garmin device was used to measure heart rate, cadence and stride length (Adams et al. 2016) which have been linked to improved running (e.g., Nicolas‐Peyrot et al. 2025). The Garmin watch provided measures of heart rate, cadence and stride length.

### 2.4 Data Analysis

Statistical analyses were conducted using SPSS software (version 30). Significance level was set at *p* = 0.05. Partial eta‐squared (*n* ^2^_*p*_) was reported as a measure of effect size, with 0.02, 0.13 and 0.26 indicating small, medium and large effect sizes, respectively (Cohen 1992). Exploratory analyses revealed no condition differences in mental fatigue, physical fatigue, sleep score from the previous night and phase of menstrual cycle at the start of the testing sessions. A series of within‐participant contrast analyses (Rosenthal and Rosnow 2010) were used first to compare the physical only warmup condition with the low and high combined warmup conditions and if significant, then second to compare the low and high combined warmup conditions.

## 3 Results

**FIGURE 2:**
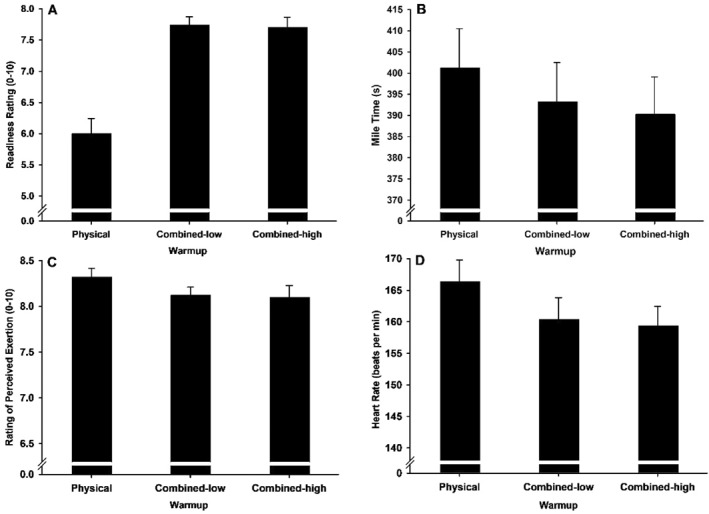
Mean (SE) readiness to perform, running time, perceived exertion and heart rate for each warmup condition.

The runners reported they were better prepared to run after the combined warmups than the physical warmup (Figure 2A). Contrast analyses confirmed that readiness ratings were higher for the combined warmups than the physical only warmup, *F*(1, 24) = 45.91, *p* < 0.001, *η*_*p*_^2^ = 0.66 and that readiness did not differ between the low and high combined warmups, *F*(1, 24) = 0.04, *p* = 0.84, *η*_*p*_^2^ = 0.00.

Participants ran 1 mile faster following combined warmups than physical only warmup (Figure 2B). Compared to the standard warmup, runners completed the mile 2.03% (8 s) quicker following the low combined warmup and 2.80% quicker (11 s) following the high combined warmup. Contrast analyses confirmed that running times were faster for combined warmups than physical only warmup, *F*(1, 24) = 25.59, *p* < 0.001, *η*_*p*_^2^ = 0.52 and that times did not differ between the low and high combined warmups, *F*(1, 24) = 1.03, *p* = 0.32, *η*_*p*_^2^ = 0.04. Sign tests compared running times between warmup conditions: most (*n* = 23, 92%) ran faster after the low combined warmup than the physical only warmup, *Z* = 3.78, *p* < 0.001 and most (*n* = 21, 84%) ran faster after the high combined warmup than the physical only warmup, *Z* = 3.73, *p* < 0.001. Finally, there was no difference between combined warmups, with 13 (52%) faster after the high than the low warmup, *Z* = 0.43, *p* = 0.67.

Ratings of perceived exertion were lower (Figure 2C) and average heart rates were slower (Figure 2D) during the time trial after combined warmups than physical only warmup. These contrast analyses showed that participants perceived less exertion after the combined warmup than physical warmup, *F*(1, 24) = 4.26, *p* < 0.05, *η*_*p*_^2^ = 0.15, whereas exertion was similar for the two combined warmups, *F*(1, 24) = 0.03, *p* = 0.86, *η*_*p*_^2^ = 0.00. Similarly, their heart rates were slower after combined than physical warmups, *F*(1, 24) = 7.30, *p* < 0.01, *η*_*p*_^2^ = 0.24, whereas heart rates were similar for the two combined warmups, *F*(1, 24) = 0.06, *p* = 0.81, *η*_*p*_^2^ = 0.00.

Running kinematics during the time trial were similar among warmup conditions. Contrast analyses found that running cadence did not differ between the physical only warmup (*M* = 178.12, SD = 8.15) and both the low (*M* = 178.72, SD = 7.27) and high (*M* = 179.52, SD = 8.43) combined warmups, *F*(1, 24) = 2.12, *p* = 0.15, *η*_*p*_^2^ = 0.09. Similarly, stride length did not differ between the physical only warmup (*M* = 1.36, SD = 0.15) and the low (*M* = 1.37, SD = 0.15) and high (*M* = 1.36, SD = 0.15) combined warmups, *F*(1, 24) = 0.18, *p* = 0.68, *η*_*p*_^2^ = 0.01.

There were no differences between mental and physical fatigue at baseline between conditions. Fatigue was then assessed after finishing the 1‐mile run. Perceived mental fatigue was higher after the physical warmup (*M* = 6.08, SD = 2.08) compared to the low (*M* = 4.16, SD = 1.84) and high (*M* = 4.72, SD = 1.67) combined warmups, *F*(1, 24) = 12.39, *p* < 0.002, *η*_*p*_^2^ = 0.34 and similar for combined warmups, *F*(1, 24) = 1.49, *p* = 0.23, *η*_*p*_^2^ = 0.06 following the time trial. Reaction times on the PFTT were faster with the physical warmup (*M* = 586, SD = 162) compared to the low (*M* = 625, SD = 239) and high (*M* = 651, SD = 243) combined warmups, *F*(1, 24) = 4.58, *p* < 0.05, *η*_*p*_^2^ = 0.20 and similar for the two combined warmups, *F*(1, 24) = 1.20, *p* = 0.29, *η*_*p*_^2^ = 0.06. Finally, perceived physical fatigue did not differ between the physical only warmup (*M* = 7.00, SD = 1.56) and both the low (*M* = 6.30, SD = 1.98) and high (*M* = 6.82, SD = 1.60) combined warmups, *F*(1, 24) = 1.80, *p* = 0.19, *η*_*p*_^2^ = 0.07.

## 4 Discussion

The present study investigated the impact of incorporating short cognitive tasks with varying levels of cognitive load into a standard physical warmup on running time trial performance, as well as associated psychological and physiological process variables. Both combined conditions improved running performance when compared with the physical only warmup. Readiness to perform increased, running time was reduced, perceived exertion was lower and heart rate was lower in the combined warmup conditions. These findings provide novel evidence that engaging executive functions during the warmup can enhance physical performance and offer a practical, easily implementable strategy for improving athletic performance. The following discussion explores these effects in greater detail.

The first study purpose was to compare the effects on running performance of a combined cognitive plus physical warmup compared to a physical only warmup. The findings revealed that adding cognitive tasks to a standard physical warmup protocol enhanced running performance by 2%–3%. Notably, there were negligible differences in running performance between the two combined warmup protocols. These findings are broadly in line with previous research (Díaz‐García et al. 2025) that described a “Goldilocks effect” in which a series of brief Stroop tasks (150–263 s task duration lasting a total of 10–17.5 min) improved sport and exercise performance whereas long Stroop tasks (a 450 s duration lasting a total of 30 min) impaired performance. The present study employed a standard 180 s task duration and used higher starting interstimulus intervals, an individualised adaptive mode and a battery of executive functions (inhibition, updating, switching) to manipulate the difficulty of the cognitive load (see O’Keeffe et al. 2019; Mortimer et al. 2024). The optimal cognitive dose must await programmatic research. It is likely that the ideal dose will depend on various factors, such as prior experience, existing cognitive fatigue and the nature of the warmup activities and protocol (Díaz‐García et al. 2025). This could vary for the same individual at different time points. The goal here would be optimal cognitive and exercise priming without inducing deleterious cognitive fatigue and 12‐min broken up into 3‐min intervals was effective for this sample and activity.

The second study purpose was to evaluate the effects of warmups on psychobiological processes. In endurance performance, cognitive‐attentional focus strategies, such as motivational self‐talk, have been shown to prolong time to exhaustion in cycling whilst reducing perceived exertion at the midpoint of exercise (Blanchfield et al. 2014). Building on this, Brick et al. (2016) proposed that effective cognitive control in endurance sport involves both reactive, stimulus‐driven processes and metacognitive regulation, enabling athletes to monitor internal and external cues and adjust pacing strategies accordingly. Consistent with this framework, the present findings show that combined warmups incorporating cognitive tasks were associated with faster running performance, lower average heart rate and reduced perceived exertion during the time trial compared with a physical‐only warmup. Although the underlying mechanisms cannot be determined from the current data, these results suggest that integrating cognitive demands into warmup routines may influence how athletes experience and regulate effort during subsequent endurance performance. From an applied perspective, combined warmups may therefore represent a practical strategy to enhance performance efficiency without increasing perceived physical demand.

The increased readiness to perform following combined warmups may be associated with differences in arousal regulation and attentional state, rather than attributed to enhanced arousal regulation and attentional readiness. Prefrontal networks, which support flexible, goal‐directed behaviour and executive control, have been proposed as a potential neural basis for changes in motor efficiency following cognitive priming (Friedman and Miyake 2021). Although neural activity was not assessed in the present study, engaging in cognitive activity or mental skills in sport has been shown to promote a performance‐oriented mindset and enhance self‐efficacy (Behncke 2004), which may contribute to perceptions of readiness and subsequent performance outcomes.

The lower average heart rate observed in the combined warmup may reflect differences in cardiac response efficiency rather than a reduction in physiological capacity. While pre‐performance anxiety and autonomic regulation were not directly measured, one possible explanation is that cognitive engagement influenced cardiovascular responses as theoretical models suggest cognitive engagement can enhance parasympathetic activity and reduce sympathetic arousal (Laborde et al. 2017). The Neurovisceral Integration Model (Thayer and Lane 2000) offers a speculative physiological interpretation, proposing that enhanced cognitive control via prefrontal networks may suppress overactive arousal circuits, thereby improving autonomic efficiency. Evidence from isometric tasks indicates that an external focus of attention can lower heart rate and improve performance (Markwell et al. 2021), whereas in running contexts, studies such as Ziv et al. (2012) report no significant differences in heart rate or running economy between external and internal focus. Pennebaker and Lightner (1980) did not measure heart rate directly, however, their findings suggest that externally oriented attention during exercise can reduce perceived fatigue and improve performance. Taken together, these observations highlight that attentional strategies may influence cardiovascular and perceptual responses in some activities, but their effects in dynamic, endurance‐based running may be context‐dependent. Given the limited research on heart rate responses following cognitive priming in sport‐specific contexts, further investigation is warranted to better understand this phenomenon.

The study measured mental and physical fatigue at the start and end of testing. Subjective mental fatigue (ratings) was lower whereas objective mental fatigue (PFTT reaction times) was higher following combined warmups than following physical warmup. This dissociation is not unprecedented as Benoit et al. (2017) found that objective fatigue can rise without corresponding subjective recognition of fatigue as feelings of fatigue and effort were unrelated in a Stroop task. More broadly, Behrens et al. (2023) argue that performance fatigue and perceived fatigue are distinct constructs, modulated by factors such as self‐regulation and motivation. This suggests that cognitive priming during warmup may increase cognitive load (reflected in objective fatigue), yet enhance focus or motivation, thus maintaining or even reducing the subjective experience of fatigue. It is likely the physical time trial also then induced further mental fatigue and participants in the cognitive conditions completed harder efforts over the mile, running faster. The physical load of the session was consistent which aligns with no condition differences observed in perceived physical fatigue throughout.

The combined warmup effects observed in this study could be partly explained by the induction of a flow‐like state (see Rautu et al. 2025). Flow is facilitated when task demands are well matched to the performer's skill level and is linked to enhanced attentional control, motivation, enjoyment and performance in sport (Csikszentmihalyi 2000; Jackson et al. 2001). The adaptive difficulty mode used in our cognitive tasks in the high combined warmup, similar to Mortimer et al. (2024) and Rautu et al. (2025), may have helped achieve this challenge–skill balance, engaging neural pathways associated with cognitive control and motor preparation reported in previous flow research (Ulrich et al. 2014; Katahira et al. 2018). Such engagement could explain the improved readiness to perform and lower average heart rates in the combined warmups, suggesting that appropriately challenging cognitive tasks may prime both psychological and physiological mechanisms for more efficient performance. Although direct evidence of flow during running is sparse, some lines of research support interpreting the findings through a flow framework. Hill et al. (2021) showed that practicing mindfulness not only improved subjective flow ratings but also was linked with improved running performance, explored through oxygen consumption. Neurophysiological investigations also show that flow involves reduced self‐referential processing and distinctive heart–brain dynamics (Khoshnoud et al. 2022), whereas recent work has suggested it possible to detect flow states via wearable sensors (Rácz et al. 2025), showing its psychobiological link. It should also be noted that in the low‐combined warmup condition, tasks were not adaptive, however, the difficulty level they were programmed at may have been well matched for the participants in this study, who were unfamiliar with the cognitive tasks used in the testing protocol. Taken together, these findings suggest that to obtain these benefits outlined above, the cognitive demands of the task must be substantial and that this should be monitored and prescribed on an individual basis. There may also be a learning and familiarisation effect so progressive overload and changing tasks over time may be necessary to continue to see performance benefits. The absence of differences in running metrics suggests that the performance benefits observed in the combined warmup conditions were unlikely to be driven by changes in gross running mechanics or maximal physiological capacity, but may instead reflect improvements in pacing strategy, movement efficiency or psychobiological factors.

### 4.1 Limitations and Future Directions

While this study provides important new insights for developing warmup protocols, demonstrating that combined physical plus mental warmups improve 1‐mile running time trial performance, interpretations of the findings should consider potential methodological limitations. Firstly, the study used a cohort of recreational runners which may limit the generalisability of the findings to other populations. Future research should aim to explore whether similar benefits are observed in athletes of different levels, particularly more competitive and elite runners and to examine whether repeated priming is needed between running intervals or whether the priming effects are sustained over longer running durations. Recreational runners may also demonstrate greater variability in performance and responsiveness to warmup interventions than highly trained athletes. While this variability may reduce measurement precision, it also reflects real‐world training populations and may partially explain the range of responses observed.

Secondly, although participants were advised to maintain consistency in factors known to influence running performance (e.g., sleep, nutrition, hydration and training load), these variables were not directly measured or strictly controlled. Greater monitoring of such variables would strengthen future investigations. Finally, the cognitive warmup protocol targeted three executive functions in combination. While this approach reflects the multifaceted nature of cognition in sport, it prevents clear identification of which specific tasks are most effective for priming. Future studies could isolate individual cognitive activities to determine their relative contributions and assess their effects across a broader range of performance outcomes. It would also be useful to explore running kinematics during each lap of the time‐trial to explore how pacing strategies may have changed and offer further insight.

#### 4.1.1 Practical Applications

The results from this study suggest that combined cognitive and physical warmups could help to optimise performance of athletes. For individuals who frequently train and perform, the results offer a practical and easily implementable method to enhance the cognitive state for short and hard endurance efforts. The complexity, intensity, order and duration of the cognitive and physical tasks within the warmup should be considered for best results and this is likely to vary based on the athlete population and change over time. The current protocol provides a starting point, however, further empirical research is needed to determine how long the effects last and whether the protocol should be altered to optimise other types of physical performance and activities (e.g., strength, skill‐based and various distances). We recommend that the cognitive and physical elements of the warmup should be intermixed for optimal results and to prevent inducing mental fatigue from sustained cognitive load (see Rautu et al. 2025). There is now justification to explore how cognitive tasks could be used in a similar way within repeated rounds of competition or tournaments to optimise performance and the associated mechanisms of these protocols (e.g., mental distraction and executive function engagement).

## 5 Conclusions

The present study revealed the performance benefits of intermixing brief cognitive tasks into warmup protocols. Both combined warmups improved running performance compared with the physical only warmup ‐ readiness to perform increased, running time was reduced, perceived exertion was lower and heart rate was slower in the combined warmups. These findings provide novel evidence suggesting that engaging executive functions, through cognitive tasks during the warmup can enhance subsequent physical performance and offers a practical, easily implementable strategy for improving athletic performance for athletes, coaches and practitioners.

## Funding

This study was supported by the Economic and Social Research Council (ES/P000711/1).

## Conflicts of Interest

The authors declare no conflicts of interest.

## Supporting information


**Table S1:** Cognitive tasks descriptions.

## Data Availability

The data that support the findings of this study are available from the corresponding author upon reasonable request.
