# Supplementary material for: Warming Up Body and Mind: Combined Cognitive and Exercise Priming Improves 1‐Mile Time Trial Performance in Recreational Runners
Source: Eur J Sport Sci. 2026 Apr 15;26(5):e70163. doi: 10.1002/ejsc.70163 (PMC13082839; doi:10.1002/ejsc.70163)
Supplement: Supplementary file 1 — Table S1: Cognitive tasks descriptions. [file EJSC-26-e70163-s001.docx]

**Supplementary Materials**

Table 1. *Cognitive Tasks Descriptions*

| **Task** | **Executive Function** | **Description** |
| --- | --- | --- |
| Colour- Shape Task | *Task Switching* | A dual rule switching task where circles and squares are presented in each trial in either red or blue. Before each trial, the task instructs participants to react based on either the 'colour' or the 'shape' of the object. In the shape condition, participants must press left for a square and right for a circle. In the colour condition, they must press left for blue and right for red. |
| Time-Load Dual Back | *Memory Updating* | A memory updating task where a series of random letters and numbers (1-9) are presented in turn. If the letter was the same as the previous letter, participants should press the ‘left’ button, if the number presented was odd participants should press the ‘1’ button and if the number was even, participants should press the ‘2’ button. |
| Colour Multi-Source Interference Task | *Response Inhibition,*  *Cognitive Interference* | A response inhibition task where 3 numbers would appear on the screen in different size fonts, one number appeared twice, and one was different. Participants were required to identify the ‘target number’ and press the corresponding button (e.g. if 131 appeared, 3 was the target number as participants should press the 3 button). The numbers were presented in various colours, but all colours should be ignored. |
| Task Switching | *Task Switching* | A multi-rule switching task where numbers between 0 and 10 flash on the screen in either red or white. For white numbers 0-5 and red odd numbers, participants should press the ‘left’ button and for white numbers 6-10 and red even numbers, participants should react by pressing the ‘right’ button. |

Mile Running Time Individual Data

| **Physical Only (seconds)** | **Low Cognitive + Physical (seconds)** | **High Cognitive + Physical (seconds)** |
| --- | --- | --- |
| 372 | 371 | 369 |
| 389 | 387 | 383 |
| 333 | 330 | 334 |
| 346 | 350 | 352 |
| 393 | 392 | 389 |
| 367 | 341 | 339 |
| 392 | 376 | 385 |
| 356 | 355 | 357 |
| 397 | 395 | 385 |
| 373 | 354 | 357 |
| 503 | 491 | 470 |
| 381 | 374 | 386 |
| 392 | 377 | 371 |
| 421 | 419 | 414 |
| 358 | 345 | 349 |
| 484 | 480 | 480 |
| 420 | 413 | 415 |
| 461 | 431 | 429 |
| 514 | 513 | 510 |
| 383 | 369 | 373 |
| 414 | 420 | 355 |
| 412 | 405 | 399 |
| 379 | 370 | 376 |
| 420 | 406 | 416 |
| 371 | 367 | 364 |
